# The Role of Artificial Intelligence (ChatGPT-4o) in Supporting Tumor Board Decisions

**DOI:** 10.3390/jcm14103535

**Published:** 2025-05-18

**Authors:** Berkan Karabuğa, Cengiz Karaçin, Mustafa Büyükkör, Doğan Bayram, Ergin Aydemir, Osman Bilge Kaya, Mehmet Emin Yılmaz, Elif Sertesen Çamöz, Yakup Ergün

**Affiliations:** 1 Department of Medical Oncology, Dr. Abdurrahman Yurtaslan Ankara Oncology Research and Training Hospital, 06200 Ankara, Turkey; cengizkaracin00@gmail.com (C.K.); mbuyukkor@hotmail.com (M.B.); ergin217aydemir@gmail.com (E.A.); osmanbkaya@gmail.com (O.B.K.); aslanim6606@gmail.com (M.E.Y.); sertesen@gmail.com (E.S.Ç.); 2 Department of Medical Oncology, Gülhane Research and Training Hospital, 06010 Ankara, Turkey; drdoganb@gmail.com; 3 Department of Medical Oncology, Bower Hospital, 21100 Diyarbakır, Turkey; dr.yakupergun@gmail.com

**Keywords:** ChatGPT-4o, tumor board, artificial intelligence, decision-making

## Abstract

**Background/Objectives:** Artificial intelligence (AI) has emerged as a promising field in the era of personalized oncology due to its potential to save time and workforce while serving as a supportive tool in patient management decisions. Although several studies in the literature have explored the integration of AI into oncology practice across different tumor types, available data remain limited. In our study, we aimed to evaluate the role of AI in the management of complex cancer cases by comparing the decisions of an in-house tumor board and ChatGPT-4o for patients with various tumor types. **Methods:** A total of 102 patients with diverse cancer types were included. Treatment and follow-up decisions proposed by both the tumor board and ChatGPT-4o were independently evaluated by two medical oncologists using a 5-point Likert scale. **Results:** Analysis of agreement levels showed high inter-rater reliability (κ = 0.722, *p* < 0.001 for tumor board decisions; κ = 0.794, *p* < 0.001 for ChatGPT decisions). However, concordance between the tumor board and ChatGPT was low, as reflected in the assessments of both raters (Rater 1: κ = 0.211, *p* = 0.003; Rater 2: κ = 0.376, *p* < 0.001). Both raters more frequently agreed with the tumor board decisions, and a statistically significant difference between tumor board and AI decisions was observed for both (Rater 1: Z = +4.548, *p* < 0.001; Rater 2: Z = +3.990, *p* < 0.001). **Conclusions:** These findings suggest that AI, in its current form, is not yet capable of functioning as a standalone decision-maker in the management of challenging oncology cases. Clinical experience and expert judgment remain the most critical factors in guiding patient care.

## 1. Introduction

Artificial intelligence (AI) is increasingly recognized as an innovative tool that supports clinical decision-making in precision oncology [1]. The rapid scientific advancements in oncology have heightened the need for technologies that facilitate evidence-based decision-making while improving time and workforce efficiency in managing complex patient care for clinicians [2]. In this context, AI-based language models have emerged as valuable assistive tools in clinical practice. GPT-based large language models (LLMs) stand out from traditional AI models due to their versatility, human-like language comprehension, and broad knowledge base [3,4]. However, they also present certain limitations, particularly in complex clinical domains such as the management of cancer patients. These limitations include the potential to generate inaccurate yet convincing responses (hallucination), lack of transparency in training processes, biases inherent in training data, and the risk of becoming outdated due to the rapidly evolving nature of medical knowledge [5,6]. These challenges must be carefully considered when employing these models as clinical decision support systems. ChatGPT is an AI model capable of answering medical questions in an evidence-based and human-like manner by accessing online resources, without the need for specialized training. Its most advanced version to date is ChatGPT-4o [1]. In the management of complex cases, particularly those involving rare tumors or patients with comorbidities, clinicians often rely on in-house or multidisciplinary tumor boards to develop the most appropriate treatment and follow-up plans. Although previous studies have investigated the role of AI in treatment planning for challenging cancer types such as head and neck cancers, thoracic tumors, and central nervous system neoplasms, these studies have generally been with limited patient populations and have not encompassed a broad range of tumor types [7,8]. For instance, Schmidl et al. reported that AI could play a supportive role in multidisciplinary tumor boards for head and neck cancers; however, the study was conducted in a limited patient cohort [7]. Similarly, Zabaleta et al. explored the potential of AI as a decision support tool in thoracic tumor boards; however, their study focused on categorized recommendations rather than detailed and open-ended treatment plans [8].

The aim of this study is to evaluate the role of artificial intelligence by comparing the treatment and follow-up plans proposed by ChatGPT-4o with those determined by an in-house tumor board for complex cancer patients with diverse tumor types. By including patients with rare tumors and comorbidities, utilizing real-world data, and assessing the performance of AI through open-ended treatment plans aligned with ESMO and NCCN guidelines, this study seeks to contribute to the existing literature.

## 2. Materials and Methods

### 2.1. Study Design and Patient Selection

This retrospective study was conducted on patients evaluated by the in-house tumor board at the Medical Oncology Department of Dr. Abdurrahman Yurtaslan Ankara Oncology Training and Research Hospital between 2023 and 2024. A total of 102 patients with complex cancer cases and complete clinical data were included in the study. Criteria for complex cases were defined as conditions that complicate the clinical decision-making process, including:

The absence of a clear treatment algorithm in standard guidelines (ESMO, NCCN), clinical scenarios requiring the evaluation of multiple treatment options (e.g., lack of response to prior therapies, the need for input from multiple disciplines in the decision-making process), high-risk profiles for standard treatment implementation due to comorbidities, and cases necessitating a personalized treatment approach (e.g., specific therapeutic needs based on the patient’s general condition or genetic/tumor characteristics).

Inclusion criteria were defined as having been evaluated by the in-house tumor board and having complete data on diagnosis, staging, treatment history, and follow-up. Patients with incomplete data were excluded from the study.

### 2.2. Patient Evaluation Form

A standardized patient evaluation form was used for the clinical assessment of patients. The form included the following information: age, sex, cancer diagnosis and stage, personal medical history from the time of diagnosis (including comorbidities, current medications, and any allergy history), family history, full reports of current systemic and local imaging studies (e.g., PET-CT, MRI), all biopsy results and surgical information and corresponding histopathological examination results since diagnosis, and complete physical examination findings. The form was designed to reflect the routine data used by the tumor board in patient management, and the same format was applied in the evaluations conducted by ChatGPT-4o (OpenAI. (13 May 2024). San Francisco, CA, USA). 

### 2.3. Tumor Board and AI Evaluation

Treatment and follow-up plans for the patients were determined through evaluation by the institutional in-house tumor board, organized and led by the Department of Medical Oncology. This in-house tumor board consisted of a core team of medical oncologists; depending on the case complexity, specialists from radiation oncology, surgical oncology, radiology, pathology, nuclear medicine, or other relevant disciplines were invited to participate. The same patients were also presented to ChatGPT-4o using the standardized patient evaluation form, and the model was asked to propose a treatment and follow-up plan in accordance with ESMO and NCCN guidelines. The prompts provided to ChatGPT-4o were structured in Turkish and formulated as follows: “Review the information in the patient evaluation form below and propose a treatment and follow-up plan in accordance with ESMO and NCCN guidelines. Present your recommendations in a detailed and open-ended manner”.

The prompts were provided without any prior training or additional guidance in order to assess the baseline performance of the model. Although it was acknowledged that providing the instructions in Turkish might influence the model’s performance in accessing English-language sources, the prompts were intentionally kept in Turkish to evaluate the model’s natural language processing capabilities in this language.

### 2.4. Evaluation Process

The treatment and follow-up plans proposed by the tumor board and ChatGPT-4o were presented, along with the patient evaluation forms, to two independent medical oncology specialists (raters) separately. The raters held board certifications in medical oncology and had a minimum of five years of clinical experience in the field. Each decision was assessed using a 5-point Likert scale (1 = strongly disagree, 2 = disagree, 3 = neutral, 4 = agree, 5 = strongly agree). During the evaluation process, patient identifiers were anonymized, and data confidentiality was strictly maintained.

### 2.5. Statistical Analysis

Data analysis was conducted using SPSS Version 27.0. Descriptive statistics were used to summarize patient demographic characteristics (age, sex, cancer diagnosis, stage, comorbidity status, and treatment setting) as well as the distribution of diagnoses.

Inter-rater reliability and the agreement between tumor board and artificial intelligence (AI) decisions were assessed using the weighted Cohen’s Kappa (κ) statistic, which is appropriate for 5-point Likert scale data. The weighted Kappa accounts for the magnitude of disagreement in ordinal data. Kappa values were interpreted as follows: κ < 0.00 (poor), κ = 0.00–0.20 (not important), κ = 0.21–0.40 (low), κ = 0.41–0.60 (moderate), κ = 0.61–0.80 (substantial), and κ = 0.81–1.00 (almost perfect).

Differences between the Likert scores assigned by the raters to tumor board and AI decisions were analyzed using the non-parametric Wilcoxon Signed Rank Test. This test is appropriate for paired samples (i.e., scores for the same patients) and ordinal data. The distribution of Likert scores was assessed using the median and interquartile range (IQR), and the appropriateness of the test was confirmed by evaluating skewness with the Shapiro-Wilk test. A significance threshold of *p* < 0.05 was adopted, and exact *p*-values were reported.

For our study, multicenter ethical approval was obtained from the Non-Interventional Clinical Research Ethics Committee of the University of Health Sciences Dr. Abdurrahman Yurtaslan Ankara Oncology Health Application and Research Center, with the approval number 2024-09/122.

## 3. Results

A total of 102 patients evaluated by the in-house tumor board between 2023 and 2024 were included in the study. The demographic characteristics of the patients and the distribution of cancer diagnoses are presented in Table 1. The most common diagnoses were breast cancer (30.4%), gastrointestinal cancers (23.5%), and lung cancer (14.7%). Among the patients, 67.6% were at the metastatic stage. Regarding ECOG performance status, 24.5% had a score of 0 and 65.7% had a score of 1. When examining comorbidity status, 48% of patients had two or more comorbidities, while 27.5% had none. The gender distribution was 55.9% female, and 71.6% of the patients were aged 50 years or older.

Inter-rater reliability and the agreement between tumor board and artificial intelligence (AI) decisions were analyzed using the weighted Cohen’s Kappa statistic; the results are summarized in Table 2. Inter-rater reliability was found to be high (κ = 0.722 for tumor board decisions, *p* < 0.001; κ = 0.794 for AI decisions, *p* < 0.001). However, agreement between tumor board and AI decisions was found to be low (κ = 0.211 for Rater 1, *p* = 0.003; κ = 0.376 for Rater 2, *p* < 0.001).

Subgroup analyses for different cancer subtypes could not be performed due to insufficient sample sizes; however, in the breast cancer subgroup, which was the only group with an adequate sample size for analysis, a statistically significant but low agreement was observed between the tumor board and ChatGPT-4o treatment decisions (Table 3).

Differences in Likert scores assigned by the raters to tumor board and AI decisions were analyzed using the Wilcoxon Signed Rank Test; the results are presented in Table 4. Rater 1 gave higher scores to tumor board decisions in 36 cases while preferring AI decisions in 7 cases (Z = 4.548, *p* < 0.001). Rater 2 rated tumor board decisions higher in 34 cases and favored AI decisions in 11 cases (Z = 3.990, *p* < 0.001). A statistically significant difference was observed between tumor board and AI decisions for both raters.

**Figure 1:**
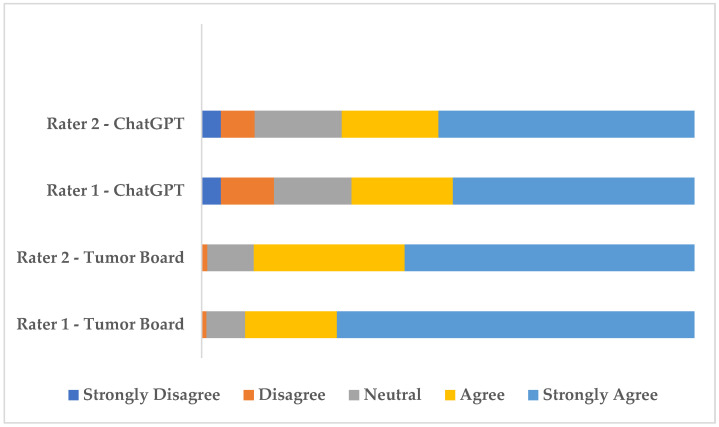
Distribution of rater ratings for tumor board and ChatGPT-4o decisions.

The distribution of rater scores is visualized in Figure 1. As shown in Figure 1, agree and strongly agree evaluations were predominant for both raters across tumor board and AI decisions. However, AI decisions received fewer “Agree” and “Strongly Agree” ratings and more “Strongly Disagree and Disagree” ratings compared to tumor board decisions, highlighting a relatively lower level of endorsement by the raters.

## 4. Discussion

This study aimed to compare the performance of ChatGPT-4o, the most recent version of ChatGPT developed by OpenAI, with institutional tumor board decisions in the treatment and follow-up planning of 102 patients evaluated by an in-house tumor board. Our findings revealed that, despite high inter-rater reliability between two independent raters, the agreement between tumor board and AI decisions was low. Both raters favored tumor board decisions over those made by the AI model, assigning higher scores to the tumor board plans in 36 and 34 cases, respectively. AI-generated decisions received fewer “Agree” and “Strongly Agree” ratings compared to tumor board decisions. Due to insufficient sample sizes, subgroup analyses could not be performed for all subgroups. However, in the breast cancer subgroup, where the sample size was adequate for analysis, a statistically significant but low agreement between the tumor board and ChatGPT decisions was observed, consistent with the findings in the overall population. These results suggest that AI, in its current form, may not be sufficient as a standalone tool in the management of complex cancer cases and that clinical experience and expert judgment continue to play a critical role in oncologic decision-making.

Numerous studies in the literature have evaluated the role of AI in oncology practice. Lukac et al. reported that ChatGPT-3.5 demonstrated lower performance in providing specific treatment recommendations for breast cancer cases compared to ChatGPT-4 [9]. Similarly, Rao et al. noted that ChatGPT-4 outperformed ChatGPT-3.5 in the diagnosis of breast cancer, demonstrating a higher level of accuracy and clinical relevance [10]. Liang et al. reported that ChatGPT-4 provided more reliable outcomes than ChatGPT-3.5 in patients with renal cancer, indicating improved consistency and clinical applicability [11]. In a study conducted on patients with sarcoma, ChatGPT-4 was shown to provide more accurate and adequate treatment recommendations compared to ChatGPT-3.5 [12]. These studies support the notion that newer versions of AI models demonstrate improved performance compared to their predecessors. In our study, the most recent version, ChatGPT-4o, was utilized; however, a low level of agreement with tumor board decisions was observed. This finding suggests that even advanced versions of AI may not yet be sufficient to fully support clinical decision-making processes in the management of complex cancer cases.

Kuş et al. examined the role of ChatGPT-4o in adjuvant treatment decision-making for patients with stage II colon cancer. They reported a moderate level of concordance between the model’s recommendations and those of clinicians; however, its alignment with NCCN and ESMO guideline recommendations was found to be low [13]. Despite the AI model having been pre-trained with medical data in that study, the observation of limited concordance even in early-stage, single-type tumors support the low level of agreement observed in our study, which involved various cancer types and clinically complex cases. In our study, the primary aim was to assess the baseline performance of the AI model without any prior training. This approach enabled an unbiased evaluation of the model’s capabilities, free from external influences or preconditioning. Zabaleta et al. reported that artificial intelligence could serve as a supportive tool in multidisciplinary thoracic tumor boards, based on a study involving 52 patients with non-small cell lung cancer [8]. However, in that study, treatment decisions were categorized, but detailed and open-ended plans were not requested from the AI model. In contrast, our study required the generation of open-ended treatment and follow-up plans, thereby allowing a more realistic assessment of the model’s applicability in clinical practice. This distinction is significant, as it highlights that while AI may serve as a supportive tool in complex cases, it is not yet capable of assuming a leading role in clinical decision-making processes.

Erdat et al., in a study involving 610 patients, reported an overall high level of concordance between multidisciplinary tumor board decisions and those generated by ChatGPT-4. However, they emphasized that this agreement was notably lower in rare and clinically complex cases [14]. Our findings are consistent with those of Erdat et al. demonstrating that AI performance remains limited in complex cases. However, in Erdat et al.’s study treatment decisions were categorized, which may lead to misleading conclusions regarding the real-world applicability of the model. In contrast, our study assessed open-ended plans for treatment and follow-up, providing a more accurate reflection of the AI model’s performance in clinical decision-making processes. Additionally, while Erdat et al. reported excellent agreement among three raters, inter-rater concordance in our study was found to be at a good level. This discrepancy may indicate that our evaluation more realistically captures differences in clinical experience and decision-making approaches among raters.

Considering that the majority of our patient population was at a locally advanced or metastatic stage, nearly half had two or more comorbidities, and the study cohort included not only common cancer types but also rare and clinically challenging malignancies such as sarcomas and head and neck cancers, the findings clearly underscore the continued necessity of clinical expertise in managing such complex cases. Kaiser et al. reported that ChatGPT was insufficient in making new treatment decisions for patients with advanced-stage colorectal cancer who had undergone multiple lines of therapy [15]. Alami et al. demonstrated that the concordance between tumor board and AI-generated decisions decreased in patients with head and neck cancer who also had comorbidities [16]. These findings support the notion that the patient profile in our study, characterized by metastatic stage disease, the presence of comorbidities, and the inclusion of rare tumor types, may have contributed to the low level of concordance observed with AI-generated recommendations.

Studies focusing on rare and complex cancer types have also highlighted the limitations of AI. Although Ammo et al. reported that ChatGPT-4o demonstrated moderate effectiveness in sarcoma patients, their study was based on only five patient scenarios, did not rely on real-world clinical data, and utilized prompt engineering techniques [17]. These factors limit the reliability and generalizability of the study. Valentini et al. also highlighted inconsistencies in ChatGPT’s responses when applied to patients with sarcoma, drawing attention to the variability and lack of robustness in the model’s clinical recommendations [18]. Luo et al. reported that ChatGPT-4 struggled to provide accurate and reliable outcomes in rare clinical cases, emphasizing the model’s limitations in handling uncommon and complex oncologic scenarios [19]. Lorenzi et al. demonstrated that ChatGPT exhibited inconsistencies, particularly in critical treatment decisions for patients with head and neck cancer, underscoring the need for cautious integration of AI into complex clinical decision-making processes [20]. These studies support the conclusion observed in our research that AI may be insufficient in managing rare and complex cases. It is evident that AI falls short in supporting clinical decision-making processes, particularly in scenarios where standard treatment guidelines are unclear or where multidisciplinary input is essential.

Our study has certain limitations. First, although we aimed to perform subgroup analyses to assess the concordance between ChatGPT-4o and tumor board treatment decisions across different cancer types, this was not feasible for subgroups other than breast cancer due to insufficient sample sizes. Weighted Cohen’s kappa requires an adequate number of cases to produce stable and interpretable results. The literature recommends a minimum of 30 to 50 cases per subgroup for valid kappa estimation [21,22]. Future studies will be designed with larger cohorts and methodologies that more closely reflect clinical practice. Second, the prompts provided to ChatGPT-4o were structured in Turkish, which may have influenced the model’s ability to access and utilize English-language medical resources. This concern is supported by findings from a study conducted with German-language prompts, in which the AI model’s performance was deemed insufficient [9]. We are currently planning follow-up studies in which queries will be submitted in English to assess potential differences in model performance related to language input. Third, our study employed a single-center, retrospective design, which may limit the generalizability of the findings and introduce selection bias. Fourth, in our study, the evaluation of ChatGPT-4o using a single prompt per case was intentionally chosen to objectively assess the model’s baseline capabilities. However, this methodology does not reflect the iterative dialogue and interactive questioning dynamics commonly employed in clinical decision-making processes. Although the literature indicates that the performance of GPT-based models can improve through interactive queries, this process was deliberately limited in the current study to ensure standardization and to minimize the risk of bias resulting from user-guided prompts [6,23]. Future studies are planned to incorporate evaluation approaches that include interactive dialogue. Fifth, the fact that the oncologists evaluating the treatment recommendations were aware of whether the decisions originated from the tumor board or ChatGPT-4o may have introduced bias, potentially favoring human-generated plans. To address this potential source of bias and enhance the reliability of future evaluations, upcoming studies will adopt a double-blind design, ensuring that evaluators remain unaware of the origin of the recommendations. Finally, differences in clinical experience among the raters may have introduced bias in the scoring of AI and tumor board-based decisions, potentially amplifying the impact of subjective interpretation on the study outcomes.

## 5. Conclusions

Our findings suggest that while AI holds potential as a supportive tool in the management of complex cancer cases, it is not sufficient as a standalone decision-maker in clinical practice. Investigating the characteristics of cases in which AI received higher scores, such as early-stage disease or tumor types with well-defined guidelines, will be important for identifying patient groups in which the model may be more effective. Moreover, the integration of AI into oncology practice requires multicenter, prospective studies with long-term follow-up. Such studies are essential to better understand how AI can be optimized in clinical decision-making, which patient populations may benefit most, and what impact AI may have on long-term patient outcomes. It is evident that in clinical practice, particularly in complex cases, the role of AI should be complemented by the expert judgment of experienced clinicians.

## Figures and Tables

**Table 1:** Demographic characteristics and distribution of diagnoses of the patients.

		Count	Column N%
Diagnosis	Lung cancer	15	14.7
	Breast cancer	31	30.4
	Genitourinary cancers	12	11.8
	Gastrointestinal cancers	24	23.5
	Head and Neck cancer	2	2
	Sarcoma	4	3.9
	Brain cancer	3	2.9
	Gynecological cancers	8	7.8
	Adrenal gland cancer	1	1
	Melanoma	1	1
	Mesothelioma	1	1
ECOG Performance Status	Score 0	25	24.5
	Score 1	67	65.7
	Score 2	4	3.9
	Score 3	6	5.9
	Score 4	0	0
Comorbidity	Lower than two	25	24.5
	Two or more	49	48
	None	28	27.5
Stage	Early stage	11	10.8
	Locally advanced stage	22	21.6
	Advanced stage	69	67.6
Treatment Line	First line	52	51
	Second or more lines	50	49
Sex	Female	57	55.9
	Male	45	44.1
Age	50 years of age and older	73	71.6
	Under 50 years of age	29	28.4

**Table 2:** Weighted Cohen’s Kappa test in all patients.

	Concordance	Cohen’s Kappa (**κ**) (Weighted)	*p*-Value	CI (95%)
Tumor Board Decisions	Rater 1–Rater 2	0.722	<0.001 *	0.60–0.85
ChatGPT Decisions	Rater 1–Rater 2	0.794	<0.001 *	0.73–0.86
Rater 1 Decisions	Konsey–ChatGPT	0.211	0.003 *	0.10–0.36
Rater 2 Decisions	Konsey–ChatGPT	0.376	<0.001 *	0.23–0.52

* *p* < 0.05.

**Table 3:** Weighted Cohen’s Kappa test in breast cancer patients.

	Concordance	Cohen’s Kappa (**κ**) (Weighted)	*p*-Value	CI (95%)
Tumor Board Decisions	Rater 1–Rater 2	0.737	<0.001 *	0.51–0.96
ChatGPT Decisions	Rater 1–Rater 2	0.744	<0.001 *	0.60–0.88
Rater 1 Decisions	Konsey–ChatGPT	0.329	0.001 *	0.11–0.55
Rater 2 Decisions	Konsey–ChatGPT	0.387	<0.001 *	0.17–0.60

* *p* < 0.05.

**Table 4:** Wilcoxon signed rank test based on rater assessments.

	Rater 1	Rater 2
Count (N)	102	102
Tumor Board > ChatGPT	36	34
ChatGPT > Tumor Board	7	11
Tied Score	59	57
Z Value	+4.548	+3.990
*p*-Value (2-tailed)	<0.001 *	<0.001 *

* *p* < 0.05.

## Data Availability

Dataset available on request from the authors.

## Abbreviations

The following abbreviations are used in this manuscript:

AI	Artificial Intelligence
NCCN	National Comprehensive Caner Network
ESMO	European Society of Medical Oncology
ECOG	Eastern Cooperative Oncology Group
IQR	interquartile range
LLMs	large language models
